# Two-step awake craniotomy for diffuse supratentorial gliomas

**DOI:** 10.1093/noajnl/vdag100

**Published:** 2026-04-11

**Authors:** Petra Bintintan-Socaciu, Shuroq Taju, Angela Elia, Benoit Hudelist, Gonzague Defrance, Elias Al Helou, Walter Thomas, Kor Gael Toruslu, Marta Garvayo, Marco Demasi, Alessandro Moiraghi, Bénédicte Trancart, Marc Zanello, Alexandre Roux, Johan Pallud

**Affiliations:** Service de Neurochirurgie, GHU-Paris Psychiatrie et Neuro­sciences, Paris, France; Institute of Psychiatry and Neuroscience of Paris (IPNP), Université Paris Cité, INSERM U1266, IMA-Brain, Paris, France; Centre de Psychiatrie et Neurosciences, Inserm, IMA-Brain, Paris, France; Department of Neurosurgery, IRCCS Ca’ Granda Foundation Ospedale Maggiore Policlinico, Milan, Italy; Department of Medical and Surgical Pathophysiology and Transplantation, University of Milan, Milan, Italy; Service de Neurochirurgie, GHU-Paris Psychiatrie et Neuro­sciences, Paris, France; Institute of Psychiatry and Neuroscience of Paris (IPNP), Université Paris Cité, INSERM U1266, IMA-Brain, Paris, France; Centre de Psychiatrie et Neurosciences, Inserm, IMA-Brain, Paris, France; Service de Neurochirurgie, GHU-Paris Psychiatrie et Neuro­sciences, Paris, France; Institute of Psychiatry and Neuroscience of Paris (IPNP), Université Paris Cité, INSERM U1266, IMA-Brain, Paris, France; Centre de Psychiatrie et Neurosciences, Inserm, IMA-Brain, Paris, France; Service de Neurochirurgie, GHU-Paris Psychiatrie et Neuro­sciences, Paris, France; Institute of Psychiatry and Neuroscience of Paris (IPNP), Université Paris Cité, INSERM U1266, IMA-Brain, Paris, France; Centre de Psychiatrie et Neurosciences, Inserm, IMA-Brain, Paris, France; Service de Neurochirurgie, GHU-Paris Psychiatrie et Neuro­sciences, Paris, France; Institute of Psychiatry and Neuroscience of Paris (IPNP), Université Paris Cité, INSERM U1266, IMA-Brain, Paris, France; Centre de Psychiatrie et Neurosciences, Inserm, IMA-Brain, Paris, France; Service de Neurochirurgie, GHU-Paris Psychiatrie et Neuro­sciences, Paris, France; Institute of Psychiatry and Neuroscience of Paris (IPNP), Université Paris Cité, INSERM U1266, IMA-Brain, Paris, France; Centre de Psychiatrie et Neurosciences, Inserm, IMA-Brain, Paris, France; Service de Neurochirurgie, GHU-Paris Psychiatrie et Neuro­sciences, Paris, France; Institute of Psychiatry and Neuroscience of Paris (IPNP), Université Paris Cité, INSERM U1266, IMA-Brain, Paris, France; Centre de Psychiatrie et Neurosciences, Inserm, IMA-Brain, Paris, France; Service de Neurochirurgie, GHU-Paris Psychiatrie et Neuro­sciences, Paris, France; Institute of Psychiatry and Neuroscience of Paris (IPNP), Université Paris Cité, INSERM U1266, IMA-Brain, Paris, France; Centre de Psychiatrie et Neurosciences, Inserm, IMA-Brain, Paris, France; Service de Neurochirurgie, GHU-Paris Psychiatrie et Neuro­sciences, Paris, France; Institute of Psychiatry and Neuroscience of Paris (IPNP), Université Paris Cité, INSERM U1266, IMA-Brain, Paris, France; Centre de Psychiatrie et Neurosciences, Inserm, IMA-Brain, Paris, France; Service de Neurochirurgie, GHU-Paris Psychiatrie et Neuro­sciences, Paris, France; Institute of Psychiatry and Neuroscience of Paris (IPNP), Université Paris Cité, INSERM U1266, IMA-Brain, Paris, France; Centre de Psychiatrie et Neurosciences, Inserm, IMA-Brain, Paris, France; Service de Neurochirurgie, GHU-Paris Psychiatrie et Neuro­sciences, Paris, France; Institute of Psychiatry and Neuroscience of Paris (IPNP), Université Paris Cité, INSERM U1266, IMA-Brain, Paris, France; Centre de Psychiatrie et Neurosciences, Inserm, IMA-Brain, Paris, France; Service de Neurochirurgie, GHU-Paris Psychiatrie et Neuro­sciences, Paris, France; Institute of Psychiatry and Neuroscience of Paris (IPNP), Université Paris Cité, INSERM U1266, IMA-Brain, Paris, France; Centre de Psychiatrie et Neurosciences, Inserm, IMA-Brain, Paris, France; Service de Neurochirurgie, GHU-Paris Psychiatrie et Neuro­sciences, Paris, France; Institute of Psychiatry and Neuroscience of Paris (IPNP), Université Paris Cité, INSERM U1266, IMA-Brain, Paris, France; Centre de Psychiatrie et Neurosciences, Inserm, IMA-Brain, Paris, France; Service de Neurochirurgie, GHU-Paris Psychiatrie et Neuro­sciences, Paris, France; Institute of Psychiatry and Neuroscience of Paris (IPNP), Université Paris Cité, INSERM U1266, IMA-Brain, Paris, France; Centre de Psychiatrie et Neurosciences, Inserm, IMA-Brain, Paris, France; Service de Neurochirurgie, GHU-Paris Psychiatrie et Neuro­sciences, Paris, France; Institute of Psychiatry and Neuroscience of Paris (IPNP), Université Paris Cité, INSERM U1266, IMA-Brain, Paris, France; Centre de Psychiatrie et Neurosciences, Inserm, IMA-Brain, Paris, France

**Keywords:** awake craniotomy, brain mapping, glioma, neurocognitive function, surgical staging

## Abstract

**Background:**

Maximal safe awake resection is the standard for diffuse gliomas, as it optimizes extent of resection while preserving functional integrity. Progressive loss of accuracy during neurocognitive testing may preclude completion of a maximal function-based resection. We assessed the prevalence, feasibility, safety, and efficacy of a 2-step awake craniotomy approach and identified predictors for requiring a second awake procedure to achieve maximal function-based resection.

**Methods:**

We conducted a retrospective single-center cohort study of 449 consecutive supratentorial diffuse glioma awake craniotomies (2009-2024). Clinical, neurocognitive, imaging, oncological, and intraoperative data were collected.

**Results:**

Among 449 awake craniotomies, 12 (2.8%) required a second awake procedure. The first surgeries were interrupted due to increasing spontaneous errors during intraoperative tests, fatigue, loss of participation, or pain-induced high blood pressure. The interval between procedures ranged from 1.0 to 7.5 months. Compared with all other awake surgeries, the second procedure showed no increase in intraoperative adverse events. Compared to the first awake procedure, the second awake procedure achieved a median additional resection rate of 32.3% (mean 38.6 ± 24.4%; range 8%-83.2%) across all patients. The proportion of complete resections increased from 0/12 to 6/12. Independent predictors of a 2-step awake craniotomy were preoperative attention impairment (*P* = .021), left-hemispheric location (*P* = .017), and insular involvement (*P* = .016).

**Conclusion:**

Two-step awake craniotomy is a rare but effective and safe strategy when the initial awake procedure must be prematurely stopped. Patients with attention deficits, left-sided lesions, or insular tumor involvement are more likely to require a 2-step awake craniotomy.

Key Points

- Two-step awake craniotomy is rare but necessary in selected cases.
- The second awake surgery is safe and significantly improves the extent of resection without increasing intraoperative adverse events.
- Specific preoperative factors predict the need for a 2-step approach such as preoperative attention impairment, left-hemispheric tumor location, and insular involvement.

Importance of the Study

In the surgical management of diffuse gliomas, achieving maximal safe resection while preserving functional integrity remains a central objective. This study provides important insights into the management of diffuse gliomas when maximal safe function-based resection cannot be completed during a single awake procedure. It demonstrates that a 2-step awake craniotomy is a safe and effective strategy when maximal resection cannot be achieved during the initial surgery and shows that repeating awake functional mapping does not increase surgical risk, while significantly improving the extent of resection. By identifying predictive factors, it enhances preoperative counseling and helps optimize patient selection.

Surgical resection is the primary treatment option for diffuse gliomas whatever the grade of malignancy, improving survival rates, epileptic seizure control, and preserving functional independence and health-related quality of life.^1–11^ Residual tumor after resection is proved to be a poor survival predictor,^10^^,^^12^^,^^13^ which highlights the importance of obtaining a maximal safe resection for diffuse gliomas, including supramarginal resection, whenever feasible.^13–15^ The aim of awake resection is to tailor the resection based on cortico-subcortical brain connectivity that forms functional boundaries in the depth of the surgical cavity.^16^^,^^17^ Nevertheless, resection is sometimes limited not only by the identification of functional, vascular, or anatomical boundaries encountered intraoperatively, but also for variable reasons: increasing fatigue or pain, insufficient intraoperative cooperation, increasing spontaneous errors during intraoperative tests not related to direct electrical stimulations, or loss of active participation.^18^^,^^19^ This can preclude the achievement of a maximal safe resection and significantly reduce resection rates.^19^ In such a situation, a second awake craniotomy can be theoretically performed to achieve a maximal safe function-based resection.

The feasibility and efficacy of such a 2-step awake craniotomy in diffuse glioma patients with a suboptimal first awake resection are poorly documented. We report a homogeneous cohort of adult patients undergoing an attempt of maximal safe function-based resection under awake conditions for a diffuse glioma. We assessed: (1) the prevalence of 2-step awake craniotomy; (2) the feasibility (including intraoperative adverse events) and the efficacy (including extent of resection) of a second awake craniotomy following a suboptimal first one; and (3) the potential predictors of the necessity of such a 2-step awake craniotomy approach.

## Methods

### Study Design and Setting

We conducted a retrospective, observational, single-center cohort study of patients operated between March 2009 and December 2024.

### Ethic Statement

The local institutional review board approved the study protocol (A01933-48), and the requirement to obtain informed consent was waived. The manuscript was written according to the Strengthening the Reporting of Observational Studies in Epidemiology checklist.^20^

### Participants

Inclusion criteria were: (1) adult patient; (2) supratentorial brain lesion; (3) maximal safe resection attempt with intraoperative functional brain mapping using direct electrical stimulations under awake condition by the same procedure; (4) histo-molecular diagnosis of grades 2-4 diffuse glioma; and (5) available preoperative and postoperative follow-up data. The study included 449 surgeries, corresponding to all eligible patients during the study period; no patients were excluded.

### Data Collection

Data gathered from medical records included: age, sex, Karnofsky performance status (KPS) score, epileptic seizures, handedness, preoperative neurocognitive evaluation (grouped into 4 neurocognitive domains: language, attention, executive functioning, and memory, as previously reported),^12^^,^^19^ tumor location, insular involvement according to the Yaşargil classification,^12^^,^^21^ tumor volume (quantified by segmentation of signal abnormalities on fluid attenuated inversion recovery (FLAIR) sequence), contrast enhancement, extent of resection and residual tumor quantified on early (<48 h) postoperative FLAIR sequence, histo-molecular diagnosis according to the 2021 WHO classification^22^ (histo-molecular reassessment for all cases diagnosed prior to September 2021), previous oncological treatment, seizure control at surgery, Awake Surgery Insufficient Cooperation (ASIC) score measured as previously reported,^19^ intraoperative side events (delayed awakening defined as a time interval ≥30 min between sedative arrest at the beginning of dura mater opening and functional mapping in a cooperative and conscious patient),^19^ epileptic seizure based on clinical assessment since electrocorticography was not systematically performed, nausea, and/or vomiting. Seizure control was defined as a patient completely free of any epileptic seizure, with or without antiseizure medication.^23^^,^^24^ The extent of resection was defined according to residual tumor volume: “total” when no residual tumor was present, “subtotal” when residual tumor was <10 cm^3^, and “partial” when residual tumor was ≥10 cm^3^.

### Surgical Procedure

Neurocognitive functions were systematically tested by a senior speech therapist before and after resection during the workup of awake resections. The tests used are summarized in Table S1. All patients underwent a structured preoperative preparation program, including dedicated consultations with the neurosurgeon and repeated sessions with a speech therapist and/or neuropsychologist, during which intraoperative tasks were rehearsed, and the awake procedure was thoroughly explained to ensure optimal understanding and cooperation.

All surgeries were performed using a reproducible “asleep-awake-asleep” protocol previously detailed.^19^^,^^24–29^ On the day of the surgery, no anxiolytic or sedative medication was administered, and antiepileptic drugs were continued as usual on an individual basis. Patient positioning on the operating table was performed prior to the induction of anesthesia, with the patient’s active participation. The patient was placed in a lateral decubitus position contralateral to the lesion, on a thick foam mattress positioned over the operating table. The head was aligned with the head-neck-trunk axis. The inferior arm was placed on a padded support in semiflexion, with the axilla positioned comfortably near the upper edge of the mattress. Particular attention was paid to ensuring a stable, comfortable, and safe position. Ambient temperature was carefully controlled, and a forced-air warming blanket was used to maintain body temperature above 36 °C. Total intravenous general anesthesia with a laryngeal mask airway was induced using propofol (target concentration of 6 mg/mL) and remifentanil (target concentration of 6 ng/mL). Pressure-controlled ventilation was applied, with respiratory pressure maintained below 15 cm H_2_O to ensure normocapnia. Intravenous antiemetics, analgesics (including paracetamol), antibiotic prophylaxis, steroids, and an intravenous loading dose of levetiracetam (500 mg) were administered at induction. The scalp was infiltrated at the incision site, the reflexion area, the head holder pins with a local anesthetic (20 mL lidocaine 2% with epinephrine).

Positive functional brain mapping was performed using direct electrical stimulations (bipolar electrode, 5-mm intertip distance; biphasic current; frequency 60 Hz; phase duration 1 ms; stimulation duration 4 s. Osiris NeuroStimulator, Inomed). After calibrating the lowest current threshold eliciting reproducible clinical responses (baseline 1 mA, 0.5-mA increment), the same stimulation parameters served for further cortical and subcortical mappings; the exposed brain was tested, and all sites were stimulated at least 3 times to confirm reproducibility of the positive functional brain mapping. Intraoperatively, all events were recorded on a clinical basis by a senior speech therapist who directly checked neurological and cognitive functions through defined and reproducible intraoperative tasks previously reported^25^^,^^30^^,^^31^ and detailed in Table S2. Sites inducing a reproducible functional impairment were marked with sterile numbered tags and considered eloquent. The lesion was resected using subpial dissection and aspiration, combined with continuous subcortical functional mapping in an awake patient performing the required tasks. Resection was stopped once the eloquent white matter fiber tracts were identified in the depth of the surgical cavity during the awake phase or when the patient committed too many errors with the occurrence of increasing spontaneous errors during intraoperative neurocognitive testing that are not induced by direct electrical stimulation, thereby making functional mapping unreliable. The residual tumor was removed under general anesthesia if considered safe from a functional point of view, but no additional resection was performed in patients for whom the resection had been stopped before successful and complete functional mapping.

When required, slight repositioning, short pauses, and local massages were performed to alleviate postural discomfort and fatigue. Thirst was managed by moistening the patient’s lips with a compress soaked in water. In the event of an intraoperative epileptic seizure, stimulations were immediately discontinued. No systemic anticonvulsant was administered; instead, the brain was irrigated with cold (4-8 °C) normal saline (0.9% NaCl) until seizure cessation. Resection was resumed once the patient was again able to perform cognitive tasks.

It is important to distinguish between 2 distinct situations: (1) resection interrupted due to unreliable functional mapping, characterized by increasing spontaneous errors not induced by direct electrical stimulation or insufficient patient cooperation, in which case resection is considered suboptimal and a second awake procedure may be proposed; and (2) resection intentionally stopped after identification of eloquent functional boundaries under reliable mapping, in which case the residual tumor is considered functionally constrained and no further resection is pursued. A second surgery was proposed only when the initial resection was clearly suboptimal due to insufficient patient collaboration, resulting in an extent of resection inferior to what was expected based on functional criteria. In such cases, a second awake procedure offered the opportunity to repeat reliable mapping with a more collaborative patient and, thereby, achieve a maximal safe, function-based resection. Conversely, in patients with partial resection but optimal and reliable functional testing during the first procedure, no second surgery was proposed, as the decision was not based on waiting for plasticity. The second surgery was performed to achieve a maximal safe function-based resection in selected cases by addressing the same craniotomy and “asleep-awake-asleep” anesthetic protocol as the first awake craniotomy; no change or adaptation has been performed between the 2 procedures. Comprehensive standardized neurocognitive assessments were not systematically performed between the 2 procedures; however, patients underwent regular clinical and task-oriented neurocognitive evaluations to ensure adequate recovery for reliable intraoperative testing before the second awake procedure.

### Statistical Analyses

The first surgeries of the 2-step awake craniotomy cohort were compared to the rest of the awake craniotomies. Descriptive statistics were reported as the mean ± SD for continuous variables and as numbers and percentage for categorical variables. Variables considered possible predictors of a second necessary awake craniotomy to achieve a maximal safe resection in univariate analysis at a *P* value < .050 were included in a multivariable logistic regression model. Unadjusted and adjusted Odds Ratios (ORs) and 95% CIs were determined for each predictor to better illustrate the impact of these factors. The level of significance was set at 0.05 for all analyses. Analyses were performed using JMP Pro software (Version 18.2.2; SAS Institute Inc.).

## Results

### Patient and Surgery Characteristics

**Figure 1.**
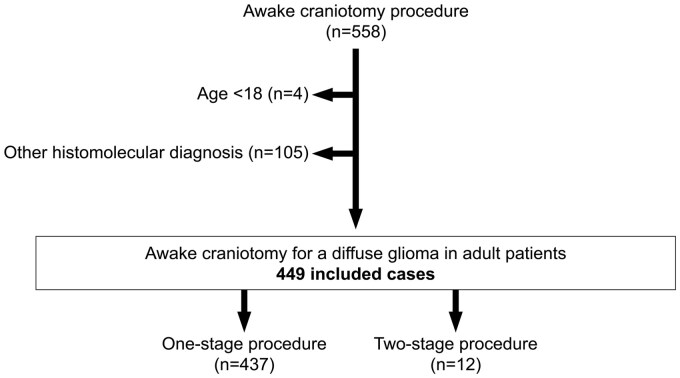
Study flow-chart.

The main characteristics of the study cohort are detailed in Table 1, and a flow chart is presented in Figure 1. Out of the 449 awake craniotomies that met the inclusion criteria, 437 (97.3%) allowed maximal function-based resection to be achieved following 1 awake procedure, and the remaining 12 cases (2.8%) required 2 awake procedures to achieve maximal function-based resection (the so-called 2-step awake craniotomy); 92% of the awake craniotomies were performed on a patient with a KPS score ≥70. At surgery, 71.3% of patients had a history of epileptic seizures, 20% of patients had a focal neurological deficit, 5.6% had signs of raised intracranial pressure, and neurocognitive impairment ranged from 37.6% to 53.4% of the studied cognitive domains.

**Table 1.** Main characteristics at surgery of the study sample (*n* = 449)

Parameters		Whole series (*n* = 449)		One-step awake surgery (*n* = 437)		Two-step awake surgery (*n* = 12)	
		*n*	%	*n*	%	*n*	%
Sex	Male	253	56.4	246	56.3	7	58.2
	Female	196	43.6	191	43.7	5	41.7
Previous oncological treatment	No	339	75.5	327	74.8	11	91.7
	Yes	110	24.5	110	25.2	1	8.3
Age at surgery (year)	Median. mean±SD (range)	42.0, 43.3 ± 13.9 (18-80)		42.0 43.3 ± 13.9 (18-80)		46.5, 44.4 ± 12.5 (23-60)	
	<42	223	49.7	222	50.8	4	33.3
	≥42	226	50.3	215	49.2	8	66.7
KPS score at surgery	Median, mean±SD (range)	100, 93.4 ± 10.3 (50-100)		100, 93.2 ± 10.4 (50-100)		100, 100 ± 0 (100-100)	
	>70	413	92.0	401	97.1	12	100
	≤70	36	8.0	36	2.9	0	0
Focal neurological deficit at surgery	No	359	80.0	347	79.4	12	100
	Yes	90	20.0	90	80.6	0	0
Raised intracranial pressure at surgery	No	424	94.4	412	94.3	12	100
	Yes	25	5.6	25	5.7	0	0
Language impairment	No	245	65.6	240	54.9	5	41.7
	Yes	204	45.4	197	45.1	7	58.3
Memory impairment	No	280	62.4	272	62.2	8	66.7
	Yes	169	37.6	165	37.8	4	33.3
Executive functioning impairment	No	209	46.6	205	46.9	4	33.3
	Yes	240	53.4	232	53.1	8	66.7
Attention impairment	No	213	47.4	211	48.3	2	16.7
	Yes	236	52.6	226	51.7	10	83.3
History of epileptic seizure at surgery	No	129	29.8	127	29.1	2	16.7
	Yes	320	71.3	310	70.9	10	83.3
Seizure control at surgery	Yes	383	85.3	371	84.9	12	100
	No	66	14.7	66	15.1	0	0
Antiseizure medication	None	103	22.9	100	22.9	3	25.0
	1	290	64.6	281	64.3	9	75.0
	≥2	56	12.5	56	12.8	0	0
Main lobar location	Frontal	205	45.7	203	46.4	2	16.7
	Temporal	101	22.5	97	22.2	4	33.3
	Parietal	66	14.7	66	15.1	0	0
	Insula^a^	70	15.6	64	14.7	6	50.0
	Occipital	3	0.7	3	0.7	0	0
	Limbic	4	0.9	4	0.9	0	0
Tumor side	Right	173	38.5	172	39.4	1	8.3
	Left	271	60.4	260	59.5	11	91.7
	Bilateral	5	1.1	5	1.1	0	0
Tumor volume FLAIR (cm^3^)	Median, mean ± SD (range)	40.4, 57.4 ± 55.9 (0.2-346.9)		40.1 57.4 ± 56.4 (0.2-346.9)		54.5, 54.5 ± 32.9 (11.7-104.1)	
	<40	223	49.7	217	49.7	6	50.0
	≥40	226	50.3	220	50.3	6	50.0
Contrast enhancement	No	234	52.1	225	51.5	9	75.0
	Yes	215	47.9	212	48.5	3	25.0
Hyperperfusion	No	226	50.3	220	50.3	6	50.0
	Yes	223	49.7	217	49.7	6	50.0
Mass effect on midline	No	362	80.8	353	81.0	9	75.0
	Yes	86	19.2	83	19.0	3	25.0
Number of cerebral lobes involved	1	226	50.3	223	51.0	3	25.0
	≥2	223	49.7	214	49.0	9	75.0
Late awakening	No	393	90.0	394	90.2	11	91.7
	Yes	44	10.0	43	9.8	1	8.3
Duration of the whole surgery (min)	Median, mean±SD (range)	223, 232 ± 67 (105-523)		223, 233 ± 68 (105-523)		214, 206 ± 35 (152-255)	
Awakening time (min)	Median, mean±SD (range)	12, 15 ± 9 (0-70)		12, 15 ± 9 (0-70)		17 19 ± 11 (7-46)	
Duration of the awake phase (min)	Median, mean±SD (range)	90, 91 ± 29 (0-245)		90, 92 ± 29 (0-245)		81, 76 ± 30 (0-110)	
Current intensity (mA)	Median, mean±SD (range)	3, 3 ± 1 (1.5-8.5)		3, 3 ± 1 (1.5-8.5)		2.5, 3 ± 1 (2-5.5)	
Intraoperative epileptic seizure	No	438	97.6	426	97.5	12	100
	Yes	11	2.4	11	2.5	0	0
Extent of FLAIR resection (%)^b^	Median, mean±SD (range)	93.2, 81.7 ± 24.2 (16.8-100)		93.7, 82.9 ± 23.9 (45.5-100)		53.8, 55.3 ± 23.8 (16.8-91.3)	
Residual FLAIR (cm^3^)^b^	Median, mean±SD (range)	3.0, 13.2 ± 23.9 (0-61.1)		2.8, 12.9 ± 23.9 (0-39.8)		15.8, 26.5 ± 22.9 (2.5-61.1)	
ASIC score	0	125	27.8	121	27.7	4	33.3
	1	191	42.5	185	42.3	6	50.0
	2	103	22.9	101	23.1	2	16.7
	3	28	6.2	28	6.4	0	0
	4	2	0.5	2	0.5	0	0
Postoperative hematoma	No	444	98.9	432	98.9	12	100
	Yes	5	1.1	5	1.1	0	0
Postoperative infection	No	442	98.4	430	98.4	12	100
	Yes	7	1.6	7	1.6	0	0

Abbreviations: ASIC, Awake Surgery Intraoperative Cooperation; FLAIR, fluid attenuated inversion recovery; KPS, Karnofsky performance status.

a Defined as A3 and 3B according to Yasargil classification.

b Based on the results of the first resection for the 2-step awake surgery subgroup.

### Two-Step Awake Craniotomies

Table 2 describes the characteristics of the 12 surgeries regarded as the first step of the 2-step awake craniotomy. None of these 12 patients had oncological treatment between the 2 awake surgeries. Reasons for interrupting resection included increasing spontaneous errors during intraoperative tests not related to direct electrical stimulations (*n* = 8), fatigue (*n* = 2), loss of active participation (*n* = 1), and increasing pain-inducing high blood pressure (*n* = 1). Intraoperative electrocorticography was performed in 8/12 patients, and no electrophysiological signs of epileptic activity were identified to account for the observed interruption of resection. The extent of resection that was stopped before the identification of eloquent connectivity defining the functional boundaries ranged from 16.8% to 91.3%. Late awakening occurred in 1 patient, and no patient experienced intraoperative epileptic seizures. Only 2 patients had an ASIC score ≥2 and were preoperatively considered at risk of poor intraoperative cooperation during the awake procedure. The time interval between the first and second awake procedures ranged between 1.0 and 7.5 months and corresponded to the recovery time for resuming preoperative neurocognitive performances. Patients underwent individualized rehabilitation, including speech and neurocognitive therapy and physiotherapy, with regular postoperative neurocognitive assessments to determine the timing of the second procedure.

**Table 2.** Characteristics of the 12 patients with a 2-step awake surgery

Patient	Sex, Age	Neurocognitive impairment	Seizure, Number of ASM	Side, location	Volume (cm^3^)	Number cerebral lobe involved	ASIC score	Intraoperative side event
1	F, 25	A	No, 0	Left, FTI	32.2	3	0	Headache
2	F, 23	E, A	Yes, 1	Left, T	29.4	1	1	
3	M, 58	L, E, Me	Yes, 1	Left, FTI	68.1	3	0	
4	F, 54	Me	Yes, 1	Right, FTI	84.3	3	1	High blood pressure
5	M, 48	L, E, A	Yes, 1	Left, T	104.1	1	2	
6	M, 31	L, E, Me, A	Yes, 0	Left, TI	10.7	2	1	Slow awakening
7	M, 56	L, E, A	Yes, 1	Left, TI	53.8	2	1	
8	M, 47	E, A	Yes, 1	Left, FP	79.0	2	1	
9	F, 46	L, E, Me, A	No, 0	Left,TI	55.2	2	0	
10	F, 45	L, A	Yes, 1	Left, TI	21.8	2	1	
11	M, 60	L, E, A	Yes, 1	Left, FTI	15.2	3	0	
12	H, 40	A	Yes, 1	Left, Fr	20.3	1	2	Poor cooperation

Patient	Current intensity for positIve mapping (mA), First surgery	Reason for arrest	Extent resection (%), (residual tumor (cm^3^) after first surgery	Immediate postoperative impairment compared to preoperative evaluation	Time interval from first stage to second stage (months)	Current intensity for positIve mapping (mA), Second surgery	Extent resection (%), (residual tumor (cm^3^) after second surgery	Histomolecular diagnosis
1	2.5	Fatigue	51.9% (15.5 cm^3^)	No	1	3.0	100% (0 cm^3^)	A4, *IDH*-mutant
2	3.5	Fatigue	91.3% (2.5 cm^3^)	L, A	4	4.0	100% (0 cm^3^)	A2, *IDH*-mutant
3	2.5	Increasing errors during picture naming test	16.8% (56.5 cm^3^)	M, L	4	2.5	100% (0 cm^3^)	A3, *IDH*-mutant
4	3.5	Increasing pain inducing high blood pressure	27.5% (61.1 cm^3^)	L, Me, A	4,5	3.0	74.5% (24.0 cm^3^)	A2, *IDH*-mutant
5	3.0	Increasing errors during picture naming test	53.1% (48.8 cm^3^)	M, L, A	5	3.0	100% (0 cm^3^)	O3, *IDH*-mutant
6	5.5	Perseverations during picture namign test, fatigue	71.9% (3.0 cm^3^)	M, L	3	5.0	100% (0 cm^3^)	A2, *IDH*-mutant
7	2.5	High rate of spontaneous erros and perseveration during picture naming test	81.8% (9.8 cm^3^)	L, A	1,5	2.5	97.9% (1.1 cm^3^)	A4, *IDH*-mutant
8	2.0	Increasing errors during picture naming test	67.0% (50.7 cm^3^)	L, A	1,5	2.0	96.9% (1.7 cm^3^)	A3, *IDH*-mutant
9	2.5	Increasing errors during picture naming test	53.2% (39.6 cm^3^)	L, E, Me, A	3.5	2.5	83.9% (8.9 cm^3^)	O2, *IDH*-mutant
10	2.0	Loss of active participation	54.5% (9.92 cm^3^)	No	2		88.5% (2.5 cm^3^)	O2, *IDH-*mutant
11	4.5	Increasing errors during picture naming test	74.0% (3.9 cm^3^)	L	7,5	3.0	82.2% (2.7 cm^3^)	O2, *IDH*-mutant
12	4.5	Difficulty in picture naming	20.8% (16.1 cm^3^)	L	1	3.5	100% (0 cm^3^)	A3, *IDH*-mutant

A: Attention functioning; A2: grade 2 Astrocytoma; A3: grade 3 Astrocytoma; Cm^3^: Cubic centimeter; E: executive functioning; F: Female; L: language; M: Male; Me: Memory functioning; O2: grade 2 Oligodendroglioma; O3: grade 3 oligodendroglioma.

### Intraoperative Findings during Second Awake Craniotomies

**Figure 2.**
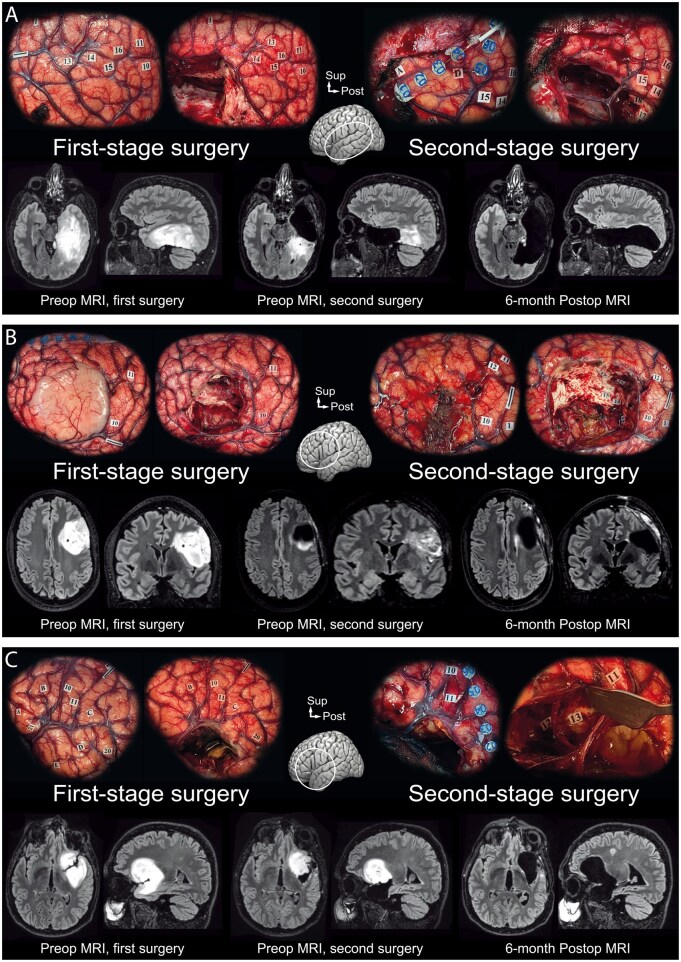
Illustrative cases. (A) A 48-year-old male (case No. 5) presenting a left temporal lesion (grade 3 oligodendroglioma) revealed initially by seizures and language impairment, undergoing a first resection. Resection stopped at the posterior limit of the lesion due to increasing spontaneous phonemic and semantic errors making the pursuit of the mapping impossible. The second resection surgery—performed 5 months later—was successful, the residual lesion being removed until reaching a posterior functional limit, encountering reading errors (17, 18) at a 3 mA stimulation intensity. (B) A 46-year-old male (case No. 8) undergoing resection for a left frontal lesion (grade 3 astrocytoma) revealed by epileptic seizures with language impairment manifestation. The first surgery was stopped at the posterior pole of the lesion, due to increasing spontaneous errors at the denomination test. The second resection surgery—performed 1.5 months later—was successful, the posterior residue of the tumor was resected until encountering language errors, with jargon expressions (13) and perseveration (14, 15) when stimulating at a 2 mA intensity. (C) A 58-year-old male (case No. 11) presenting with a left fronto-temporo-insular lesion (grade 2 oligodendroglioma) revealed by epileptic seizures. The first resection was limited after the removal of the temporal and insular portions of the lesion by increasing spontaneous errors at the denomination test, which made mapping impossible. The second resection surgery—performed 7.5 months later—was successful; the residual frontal portion of the tumor was removed, reaching a functional limit, by identifying semantic paraphasia (12, 13) when stimulating at a 3 mA intensity.

The duration of the whole surgery was shorter in patients of the second surgery of the 2-step awake craniotomies compared to patients of the rest of the awake craniotomies (189 ± 51 min vs 233 ± 62, *P* = .009). The incidence of intraoperative adverse events did not differ in patients of the second surgery of the 2-step awake craniotomies compared to patients of the rest of the awake craniotomies: high blood pressure (*n* = 1/12, 8.3% vs *n* = 24/437, 5.5%; *P* = .672), late awakening (*n* = 0/12, 0% vs *n* = 45/437, 10.3%; *P* = .109), intraoperative insufficient cooperation (*n* = 0/12, 0% vs *n* = 30/437, 6.9%; *P* = .195), headaches (*n* = 1/12, 8.3% vs *n* = 66/437, 15.1%; *P* = .485), repositioning during awake phase (*n* = 0/12, 0% vs *n* = 8/437, 1.8%; *P* = .508), and intraoperative epileptic seizures (*n* = 0/12, 0% vs *n* = 11/437, 2.52%; *P* = .437). In no case did the patient’s insufficient intraoperative cooperation, increasing spontaneous errors during intraoperative tests not related to direct electrical stimulations, fatigue, or loss of active participation lead to the arrest of the awake functional mapping. In all cases, intraoperative brain mapping allowed identifying eloquent white matter fiber tracts in the depth of the surgical cavity. No surgery-related complication was reported. Illustrative cases are represented in Figure 2. Following the second surgery of the 2-step awake craniotomies, the extent of resection ranged between 66.1% and 100% (median 98.5, mean 89.8% ± 12.1%), and the residual tumor ranged between 0 and 23.9 cc (median 0.6, mean 3.4 ± 6.9 cc). Compared to the first awake procedure, the second awake procedure achieved a median additional resection rate of 32.3% (mean 38.6% ± 24.4%; range 8%-83.2%) across all patients. The proportion of complete resections increased from 0/12 to 6/12.

In these patients, no surgical site infection, hematoma requiring surgical evacuation, or systemic complication occurred.

### Predictors of 2-Step Awake Craniotomies

In multivariable analysis (Table 3), preoperative attention impairment (aOR: 4.89; 95% CI, 1.24-32.39; *P* = .021), tumor location within the left hemisphere (aOR: 7.15; 95% CI, 1.35-132.15; *P* = .017), and insular involvement (aOR: 4.31; 95% CI, 1.30-16.61; *P* = .016) were independent predictors for a 2-step awake craniotomy. All the remaining factors showed no significant influence on the study cohort.

**Table 3.** Risk factors of a 2-step awake surgery

Parameters		Two-step awake surgery Adjusted Odds Ratio*					
		Unadjusted Odds Ratio			Adjusted Odds Ratio*		
		uOR	CI95%	*P* value	aOR	CI95%	*P* value
Sex	Female	1 (ref)					
	Male	1.09	0.34-3.72	0.880			
Previous oncological treatment	No	1 (ref)					
	Yes	0.27	0.02-1.44	0.146			
Age at surgery (year, median)	<42	1 (ref)					
	≥42	2.07	0.64-7.82	0.228			
KPS score at surgery	>70	1 (ref)					
	≤70	NE					
Focal neurological deficit a surgery	No	1 (ref)					
	Yes	NE					
Raised intracranial pressure at surgery	No	1 (ref)					
	Yes	NE					
Preoperative language impairment	No	1 (ref)					
	Yes	1.71	0.53-5.84	0.364			
Memory impairment	No	1 (ref)					
	Yes	0.82	0.22-2.66	0.753			
Executive functioning impairment	No	1 (ref)					
	Yes	1.76	0.55-6.69	0.346			
Attention impairment	No	1 (ref)			1 (ref)		
	Yes	4.67	1.21-30.59	**0.023**	4.89	1.24-32.39	**0.021**
History of epileptic seizure et surgery	No	1 (ref)					
	Yes	2.04	0.53-13.45	0.320			
Seizure control at surgery	No	1 (ref)					
	Yes	NE					
Antiseizure medication	No	1 (ref)					
	Yes	0.89	0.26-4.07	0.864			
Main lobar location	Frontal	1 (ref)					
	Temporal	4.19	0.75-23.24	0.089			
	Parietal	NE					
	Insula	9.51	0.87-48.31	0.063			
	Occipital	NE					
	Limbic	NE					
Tumor side	Right	1 (ref)			1 (ref)		
	Left	7.27	1.39-133.56	**0.014**	7.15	1.35-132.15	**0.017**
	Bilateral	NE					
Tumor volume FLAIR (cm^3^, median)	<40	1 (ref)					
	≥40	0.98	0.30-3.20	0.981			
Contrast enhancement	No	1 (ref)					
	Yes	0.54	0.14-1.73	0.301			
Hyperperfusion	No	1 (ref)					
	Yes	1.01	0.31-3.29	0.981			
Mass effect on midline	No	1 (ref)					
	Yes	1.42	0.31-4.87	0.617			
Insula involved	No	1 (ref)			1 (ref)		
	Yes	4.83	1.49-18.35	**0.008**	4.31	1.30-16.61	**0.016**
Number of cerebral lobes involved	1	1 (ref)					
	≥2	3.13	0.92-14.23	0.069			
Late awakening	No	1 (ref)					
	Yes	1.83	0.27-7.24	0.472			
Intraoperative epileptic seizure	No	1 (ref)					
	Yes	NE					
ASIC score	0	1 (ref)					
	1	0.98	0.27-3.55	0.977			
	2	0.59	0.11-3.34	0.549			
	3	NE					
	4	NE					

Unadjusted and adjusted odds ratios by logistic regression model (449 patients, 12 with a 2-step awake surgery). Significant *p*-values are highlighted in bold.

Abbreviations: ASIC, Awake Surgery Intraoperative Cooperation; FLAIR, fluid attenuated inversion recovery; KPS: Karnofsky performance status; NE, not estimable (zero event); OR, odds ratio.

## Discussion

### Key Results

In this retrospective, monocentric cohort study, including 449 consecutive awake craniotomies for a diffuse supratentorial glioma, we show that a 2-step awake craniotomy to achieve a maximal safe resection was (1) a rare event, occurring in 2.8% of cases; (2) caused mostly by increasing spontaneous errors during intraoperative tests not related to direct electrical stimulations during the first awake craniotomy; (3) predicted by a preoperative attention impairment, a left hemisphere tumor location, and an insular involvement; and (4) successful in all cases in terms of maximal function-based resection, brain connectivity being identified in the depth of the surgical cavity, and extent of resection being improved compared to the first surgery.

### Interpretation

Repeated awake craniotomies for a recurrent diffuse glioma have already been reported as safe and efficient procedures, with acceptable intraoperative seizure rates and perioperative tolerability.^29^^,^^32–41^ Previous studies focused on recurrent diffuse gliomas and highlighted brain plasticity and a sufficient time interval between the 2 surgeries as main factors allowing for repeated resections.^34^ Louppe et al^35^ reported a single case of a 29-year-old patient who experienced intractable seizures and severe language disorders due to a large left fronto-temporo-insular tumor. They performed a first awake procedure with “initial laborious language mapping” allowing a partial debulking, and a second awake procedure 3 months later following neurocognitive improvement to achieve a maximal safe function-based resection. Although their case differed from the present series by the presence of significant preoperative neurocognitive disorders that predicted a difficult first awake resection, it suggested that the 2-step awake craniotomy method could offer the possibility of obtaining maximal safe resection that preserves brain connectivity. Here, we assessed 2-step awake surgeries for the same glioma incompletely removed during the first awake craniotomy due to progressive inability to perform the intraoperative neurocognitive tasks. The patients benefited from a recovery and rehabilitation time between the 2 procedures to ensure a return to the baseline neurocognitive status before the second awake craniotomy. The short time between the 2 procedures allowed early functional compensation mechanisms but no long-term glioma-related brain plasticity. Although postoperative functional reorganization has been suggested as a mechanism facilitating more extensive resection during repeat awake surgery in selected cases, our study was not designed to assess this hypothesis, and the relatively short interval between procedures in our cohort mainly reflected clinical and neurocognitive recovery rather than an intentional strategy to promote functional plasticity.

Among the factors influencing the necessity of 2-step awake craniotomy in our study, the insular location of a diffuse glioma proved to be predictive. The increased use of staged awake procedures in selected insular gliomas under study (types 3A and 3B according to Yaşargil) likely reflects the functional complexity of transopercular approaches, where prolonged and demanding intraoperative testing through eloquent but non-invaded cortex may challenge task reliability, rather than a limitation of the surgical technique itself.^29^^,^^37^ The importance of achieving a maximal safe resection for insular tumors was already highlighted by Ribeiro et al,^37^ who demonstrated that a large resection after the first surgery candidates the patient for a successful second surgery in case of recurrence. Demasi et al^29^ demonstrated that following awake craniotomy for a recurrent insular diffuse glioma, there was no increase in adverse events or complications compared to the initial awake craniotomy. In our series, the second surgery always led to a larger resection and was therefore considered successful. There is no previous evidence that left hemispheric location and attention deficit on preoperative evaluation might increase the risk of incomplete removal during the first awake craniotomy due to a progressive inability to perform intraoperative neurocognitive tasks. However, lesions located within the dominant hemisphere for language are more frequently addressed with awake mapping. In this context, function-based resection is more likely to induce intraoperative language disturbances, which may prematurely interrupt the awake procedure before the identification of eloquent boundaries. Moreover, perturbations of the superior longitudinal fasciculus, known to subserve attentional networks, may contribute to transient attention deficits during mapping, further compromising task reliability. This consideration is particularly relevant even though our center routinely performs awake mapping for right hemispheric diffuse gliomas as well. Similarly, attention deficit may induce increasing spontaneous errors during intraoperative tests not related to direct electrical stimulations, leading to the arrest of the awake procedure before the identification of eloquent boundaries. We therefore consider that preoperative attention impairment should not be considered a contraindication to awake surgery but rather as a marker of increased risk for intraoperative task fatigue and reduced reliability of functional mapping, thereby supporting tailored surgical strategies and informed preoperative counseling.

### Generalizability

Strengths of this study included: (1) the homogeneous and large population of adult patients harboring diffuse gliomas who underwent the same awake procedure; (2) the standardized data collection; and (3) the presence of a control group of patients undergoing a unique maximal safe awake resection. By characterizing preoperatively the clinical and neurocognitive profiles of patients requiring 2-step procedures, the present study could help: (1) preoperatively counsel patients of a rare but existing risk of requiring 2 awake procedures to achieve maximal safe resection of their gliomas, particularly in patients who present with a left insular diffuse glioma and with attention neurocognitive deficits; (2) increase patients’ adherence to the surgical treatment and facilitate their collaboration during the awake phase of the surgeries; and (3) improve patient selection suitable for a 2-step resection for their glioma.

### Limitations

Limitations include the retrospective and monocentric design and the absence of an external validation cohort. We acknowledge that (1) the potential bias induced by the inclusion of patients who had previously received oncological treatment, which was controlled by the inclusion of this parameter in multivariable analyses; (2) the second surgery of the 2-step awake craniotomies was included in the control group to maintain a stable population sample; (3) the restricted statistical power related to the small number of 2-step awake craniotomies in this population that limited the significance of multivariable analyses, as reflected by broad CIs; and (4) the diagnosis of seizures and their characterization were based on a clinical basis only without the systematic use of electroencephalography, which may have helped identify subclinical epileptic activity potentially contributing to errors during neurocognitive testing. These findings should be interpreted with the full knowledge of these limitations and should be validated with external large databases.

## Conclusion

Two-step awake craniotomy for obtaining a maximal safe resection in supratentorial diffuse glioma patients is a safe and effective procedure that should be performed when the initial resection must be stopped for increasing spontaneous errors during intraoperative tests not related to direct electrical stimulations. Patients with a left hemispheric diffuse glioma, an insular location, and a preoperative attention deficit are the most probable candidates for a 2-step awake craniotomy. This study reinforces the concept that repeated awake surgeries are feasible, safe, and clinically valuable, provided that patient selection and perioperative neurocognitive management are carefully optimized.

## Supplementary Material

vdag100_Supplementary_Data

## Data Availability

All datasets analyzed in this study are available from the corresponding author on reasonable request.
