# Supplementary material for: Two-step awake craniotomy for diffuse supratentorial gliomas
Source: Neurooncol Adv. 2026 Apr 11;8(1):vdag100. doi: 10.1093/noajnl/vdag100 (PMC13200787; doi:10.1093/noajnl/vdag100)
Supplement: vdag100_Supplementary_Data [file vdag100_supplementary_data.docx]

**Supplementary Table 1.** Neurocognitive evaluations.

| **Domain** | **Test name** |
| --- | --- |
| Handedness | Edinburgh handedness inventory |
| Level of Education | Barbizet scale |
| Health-related quality of life | EORTC QLQ C30  EORTC QLQBN20 |
| Attention | Trail Making Test, part A  D2 test of attention  Dual task paradigm of Baddeley  Paced Auditory Serial Audition test (PASAT) |
| Working memory | Forward and backward verbal span, Wechsler Adult Intelligence Scale (WAIS) IV  Forward and backward digit span, MEM III  Dual task paradigm of Baddeley  Paced Auditory Serial Audition test |
| Executive functions | Stroop test  Go no go test, Frontal Assessment Battery (Batterie Rapide d’Efficience Frontale, BREF)  Trail Making Test, part B and B-A  Rey figure copying test |
| Praxis | Rey figure copying test |
| Episodic memory | 5 words test, Montreal Cognitive Assessment (MoCA)  Delayed Rey figure copying test |
| Social cognition | Emotion recognition, Pictures of Facial Affect of Ekman  Direct and indirect speech acts interpretation, Montréal Evaluation de la Communication de poche (MEC-P) protocol |
| Language | Picture naming, Dénomination Orale 80 (DO80)  Literal and categorical word fluencies  Semantic association task, Batterie d’Evaluation des Troubles Lexicaux (BETL)  Pyramid and Palm Tree Test (PPTT) Boston Diagnostic Aphasia Examination (BDAE) |

**Supplementary table 2**. Intraoperative identification of the white matter fiber tracts defining the deep functional boundaries of the glioma resection using intraoperative brain mapping under awake condition according to our practice.

|  | |  |  | |  | |  |
| --- | --- | --- | --- | --- | --- | --- | --- |
| **White matter fiber tracts** | **Functional role screened to identify intraoperatively the fiber tract** | | | **Tasks performed during awake surgery** | | **Functional responses elicited by direct electrical stimulations** | |
| **Corticospinal tract**  **Right**  **left** | Primary motor activity  Primary motor activity | | | None specific  None specific | | Involuntary movement  Involuntary movement | |
| **Thalamocortical tract**  **Right**  **Left** | Primary sensory activity  Primary sensory activity | | | None specific  None specific | | Sensory disturbances  Sensory disturbances | |
| **Visual pathways**  **Right**  **Left** | Vision  Vision | | | None specific  None specific | | Vision disturbances  Vision disturbances | |
| **Uncinate and inferior fronto-occipital fasciculi complex**  **Right**    **Left** | Visual semantic cognition  Mentalizing  Basic emotion recognition  Spatial cognition  Verbal semantic cognition  Orthographic processes | | | Pyramid and palm tree test  Modified reading the mind in the eyes test  DO80 naming test  Pyramid and palm tree test | | Semantic paraphasia  Verbal perseveration  Syntactical errors  Visual semantic error  Emotion recognition impairment  Spatial neglect | |
| **Arcuate and superior longitudinal fasciculi complex**  **Right**  **Left** | Mentalizing  Basic emotion recognition  Reading  Speech articulation  Speech production  Phonological working memory  Phonological processing  Verbal motor planning | | | Modified reading the mind in the eyes test  DO80 naming test | | Emotion recognition impairment  Anarthria/dysarthria  Repetitions errors  Alexia  Phonological paraphasia  Anomia | |
| **Fronto-striatal tract**  **Right**    **Left** | Motor initiation  Bimanual coordination  Speech programming  Motor initiation  Bimanual coordination  Speech programming | | | DO80 naming test  Motor actions  DO80 naming test  Motor actions | | Motor inhibition  Speech arrest  Motor inhibition  Speech arrest  Verbal perseveration | |
| **Frontal aslant tract**  **Right**  **Left** | None specific  Speech programming | | | None specific  DO80 naming test | | None specific  Speech arrest  Verbal perseveration | |
